# Cross-cultural adaptation, validity, reliability and responsiveness of the Japanese version of the Victorian Institute of sports assessment for patellar tendinopathy (VISA-P-J)

**DOI:** 10.1186/s13102-023-00615-5

**Published:** 2023-01-11

**Authors:** Ishin Togashi, Masashi Nagao, Hirofumi Nishio, Shojiro Nozu, Yuki Shiota, Yuji Takazawa

**Affiliations:** 1 Department of Sports Medicine and Sportology, Graduate School of Medicine, Juntendo University, Tokyo, 113-0033 Japan; 2 Department of Sports Medicine, Juntendo University, Tokyo, Japan; 3 Innovative Medical Technology Research & Development Center, Juntendo University, Tokyo, Japan; 4 Institute of Health and Sports Science & Medicine, Juntendo University, Chiba, Japan

**Keywords:** COSMIN, Japanese, Patellar tendinopathy, Validation study

## Abstract

**Background:**

This study aimed to translate, adapt, and test the psychometric properties of the Japanese version of the Victorian Institute of sports assessment for patellar tendinopathy (VISA-P-J).

**Methods:**

This prospective cohort study registered 43 participants ≥ 18 years old with a history of painful symptoms in the inferior pole of the patella to the proximal patellar tendon lasting ≥ 1 month for patellar tendinopathy. Pain in daily life and during sports activities, symptom classification, and patient global impression of change were assessed at the baseline, 1 week, and 12 weeks. The psychometric properties, test–retest reliability, standard error of measurement, internal consistency criterion validity, construct validity, responsiveness, and interpretability, of the VISA-P-J were calculated according to the COSMIN.

**Results:**

The two-way random-effects, absolute agreement intraclass correlation coefficient for test–retest reliability of VISA-P-J was 0.87 (95% confidence interval: 0.78, 0.93), and the standard error of measurement of VISA-P-J was 0.89. The Cronbach's alpha for internal consistency of VISA-P-J was 0.81. A correlation between VISA-P-J and Roel’s classification, Visual Analog Scale for pain (VAS)-Active Daily Living, and VAS-Sports (r = − 0.52, r = − 0.66, r = − 0.86, *p* < 0.01, respectively) was observed for criterion validity. All hypotheses of the hypothesis-testing method to evaluate construct validity and responsiveness of VISA-P-J were substantiated. The minimal clinically important difference of VISA-P-J was 7 points.

**Conclusion:**

We demonstrated that the VISA-P-J was a reliable, valid, and responsive assessment method for individuals with chronic pain in the patellar tendon.

**Supplementary Information:**

The online version contains supplementary material available at 10.1186/s13102-023-00615-5.

## Background

Patellar tendinopathy is a disorder of the patellar tendon and, is histopathologically characterized by the development of structural degeneration of tendon tissue [1]. The degenerative change is typically caused by overloading of normal tendons, and progresses in the order of reactive tendinopathy, tendon dysrepair, and degenerative tendinopathy which is refferred as a continuum model [2]. This injury generally occurs in individuals who engage in sports and recreational activities that require sudden acceleration and deceleration, such as jumping, climbing, and kicking [3]. The prevalence of patellar tendinopathy has been high in sports; about 45% and 32% of volleyball and basketball players, respectively, have been reported to have patellar tendinopathy [3]. The typical symptoms of patellar tendinopathy are chronic and persistent pain, which negatively affects athletes’ sports careers [4, 5]. Therefore, early and accurate evaluation and appropriate interventions are required.

The pathophysiology of a disease and the effectiveness of treatment are generally assessed using objective and subjective evaluations. Patient-reported outcome measures (PROMs) have been widely used for subjective assessments. Due to the limitation of understanding patients’ pain and daily function with objective assessment, combination with subjective evaluation has an advantage [6].

The Victorian Institute of Sport Assessment Scale for Patellar Tendinopathy questionnaire (VISA-P) was developed [7], and has been translated into languages appropriate for different regions and validated in Spanish, Brazilian Portuguese, and others [8–19]. It is widely used in clinical and sports settings to assess the subjective symptoms for patellar tendinopathy in many countries. However, a systematic review reported low-to-moderate reliability despite high validity and responsiveness in Spanish, Brazilian Portuguese, and Dutch versions [20]. Although ensuing psychometric properties, such as reliability and validity, are fundamental, the Japanese version of the VISA-P (VISA-P-J) has not yet been evaluated in the Japanese population.

The COnsensus-based Standards for the selection of health Measurement INstruments (COSMIN) has been used to develop and validate PROMs. The COSMIN guidelines include establishing terms, definitions, and standard criteria for the psychometric property [21, 22]. Although complying with the COSMIN guidelines is recommended to clarify the psychometric properties of PROMs, no study of the VISA-P followed its development. In addition, other than the studies on VISA-P’s Spanish translation [8, 23], no reports have covered its reliability, validity, responsiveness, and interpretability. This study aimed to translate, adapt, and test the psychometric properties of the Japanese version of the Victorian Institute of sports assessment for patellar tendinopathy (VISA-P-J) using the COSMIN.

## Methods

### Participants

Written consent to participate in this study was obtained from all participants, with no financial incentives. The inclusion criteria were as follows: (1) pain at the inferior pole of the patella that had persisted for ≥ 1 month for patellar tendinopathy, (2) being > 18 years old, and (3) the ability to provide written informed consent. The exclusion criteria were as follows: (1) inability to understand Japanese language, (2) history of knee surgery or surgery during the study period, and (3) other knee injuries that can mask the anterior knee pain. The participants were recruited at only the Athletic training room at the Juntendo University. We examined MRI or US as required by physician to confirm lesions and clinical conditions.

### Study design

This study was performed at Juntendo University, Chiba, Japan, from February 2021 to February 2022, with ethical approval from the institutional review board of the Research Ethics Committee of Juntendo University (H20-0289). Informed consent to participate in this study was obtained from all participants, with no financial incentives. Evaluations were made at three time points: at the baseline, 1 week, and 12 weeks. The observation items were the VISA-P-J, visual analog scale (VAS) for pain, Roel's classification of symptoms [24], and Patient Global Impression of Change (PGIC) [25]. The VISA-P-J, VAS, and Roel’s classification were used during the study period. The PGIC was assessed at 12 weeks. The participants neither received any interventions during the study period nor were they advised any activity restrictions. The sample size was determined based on COSMIN’s recommendation.

### Translation procedures of the Japanese of version of VISA-P

We developed the VISA-P-J based on a guideline for cross-cultural adaptation of self-report measures [26]. The process was composed of the following six steps: (1) Translation of the original English version into Japanese; (2) Review and modification of the translated items by experienced sports medicine physicians; (3) Back translation into English by an English native speaker; (4) Review of the back-translated version by experienced sports medicine physicians and sports medicine specialists; (5) Testing of the prefinal version on a group of 43 people; and (6) Approval of the final version of the VISA-P-J (Additional file 1).

### Measurement instruments

The participants’ pain was assessed using the VAS. The classification according to symptoms was based on the four categories of symptoms developed by Roels et al. [24, 27]. The PGIC was used to evaluate participants’ subjective judgment about any change (improvement) perceived [25]. It comprised a 7-point scale that indicated the following: 1 = very much worse, 2 = much worse, 3 = minimally worse, 4 = no change, 5 = minimally improved, 6 = much improved, and 7 = very much improved.

### Psychometric properties

We evaluated the reliability, validity, responsiveness, and interpretability of the VISA-P-J according to the COSMIN guidelines [21, 28]. The test–retest reliability measures the stability of a stable construct questionnaire obtained from the same population on two occasions. A retest was performed one week after the primary test to avoid changes in participants’ condition between tests and recall bias [29]. Measurement errors were calculated using standard error of measurement (SEM). Internal consistency, which measures whether subscales measure the same concept, was evaluated using Cronbach's alpha [30].

Clarifying the aim of measurement, target population, and concepts [30] was required to evaluate content validity. For the VISA-P-J, we set a measurement aim to assess the pain and disability of patellar tendinopathy subjectively. This instrument was used to assess the symptoms, function, and ability of participants with patellar tendinopathy [7, 30]. To assess criterion validity, which measures whether a scale is related to existing external criteria, we assessed the correlations between the VISA-P-J score and (1) Roel’s classification, (2) VAS–activities of daily living (VAS-ADL), and (3) VAS-Sports as the criterion validity. Construct validity, which indicates whether a scale represents the intended construct, and responsiveness were assessed using hypothesis testing [30]. The construct validity hypotheses were H1–H3, and the hypotheses of responsiveness were H4–H6. The hypothesis testing method was proven when at least 75% of the predetermined hypothesis was achieved [30].

#### H1

VISA-P-J scores will have a negative correlation with symptom classification.

#### H2

VISA-P-J scores will have a negative correlation with the VAS-ADL scores.

#### H3

VISA-P-J scores will have a negative correlation with the VAS-Sports scores.

#### H4

Participants who answer 5–7 on the PGIC scale will have a more positive change in VISA-P-J scores than those who answer 1–4 on the PGIC scale.

#### H5

The amount of change in VISA-P-J scores will negatively correlate with the amount of change in VAS-ADL scores.

#### H6

The amount of change in VISA-P-J scores will negatively correlate with the amount of change in VAS-Sports scores.

The floor or ceiling effect was defined as > 15% of participants reporting minimum or maximum scores, respectively [30]. The minimal clinically important difference (MCID) was defined as the participant’s perception of whether there was an important change in the patellar tendinopathy status [31]. The within-group MCID was measured according to the mean score changes in the PGIC scale between "minimally improved" and "no change" from baseline to 12 weeks.

### Statistical analysis

The demography of the participants was shown with standard deviation (SD). Test–retest reliability was assessed using two-way random-effects, absolute agreement intraclass correlation coefficient (ICC). ICC values less than 0.5 are indicative of poor reliability, values between 0.5 and 0.75 indicate moderate reliability, values between 0.75 and 0.9 indicate good reliability and values greater than 0.90 indicate excellent reliability [32]. Criterion validity, construct validity (H1–H3), and responsiveness (H5–H6) were assessed using Pearson's and Spearman's rank correlation coefficient tests. Responsiveness (H4) was tested using the Mann–Whitney U test. All analyses were performed using SPSS version 22 for Windows (SPSS Inc., Chicago, IL, USA), with statistical significance set at *p* < 0.05.

## Results

### Participants

Forty five participants were initially recruited based on the inclusion criteria. Two participants were withdrawn on account of unreachability; finally, 43 were included in this study. The participants’ characteristics are presented in Table 1. The duration of participants' symptoms onset ranged from 1 to 136 months, with a mean ± SD of 23 ± 27 months. In the Roel’s classification, fifteen participants were in stage 1, 24 in stage 2, 4 in stage 3, and 0 in stage 4.

**Table 1:** Characteristics of Participants (*N* = 43)

Characteristic	Mean ± SD
Mean age, years	20.2 ± 2.6
Sex (Male/Female)	23/20
Mean height, cm	169.8 ± 10.8
Mean weight, kg	67.0 ± 14.7
Mean BMI	23.1 ± 3.7
Side of injury (Right/Left)	22/21

*SD* Standard deviation; *BMI* Body mass index

### Psychometric properties

The ICC for test–retest reliability (95% confidence interval, CI) was 0.87 (0.78–0.93), and the SEM was 0.89. Cronbach's alpha for internal consistency was 0.81 (0.71–0.88) (Table 2).

**Table 2:** Measurement property of the VISA-P-J

	VISA-P-J
ICC (95% CI)	0.87 (0.78–0.93) **
Baseline, mean (95% CI)	74.3 (69.0–79.5)
1 week, mean (95% CI)	74.0 (68.4–79.5)
12 weeks, mean (95% CI)	77.1 (71.8–82.5)
Time interval (Baseline–1 week), mean days (95% CI)	7.0 (6.8–7.1)
SEM	0.89
Cronbach’s alpha (95% CI)	0.81 (0.71–0.88)
*Correlation with the VISA-P-J*	
Roel's classification, r	− 0.52 **
VAS-ADL, r	− 0.66 **
VAS-Sports, r	− 0.86 **
*Correlation with amount of change of the VISA-P-J* *(Baseline–12 weeks)*	
Amount of change of the VAS-ADL, r	− 0.52 **
Amount of change of the VAS-Sports, r	− 0.76 **
Floor effects (%)	0
Ceiling effects (%)	14.0
MCID	7

*VISA-P-J* Japanese version of the Victorian Institute of sports assessment scale for patellar tendinopathy; *ICC* intraclass correlation coefficient; *SEM*, standard error of measurement; *VAS-ADL* visual analog scale—activities of daily living; *VAS-Sports*, visual analog scale—sports; *PGIC* patient global impression of change; *MCID* minimal clinically important difference. *95% CI* 95% confidence interval

**p < 0.01

The results for criterion validity and construct validity are respectively shown in Table 2. The VISA-P-J showed a correlation between symptom classification, VAS-ADL, and VAS-Sports, respectively (r = − 0.52, r = − 0.66, r = − 0.86, *p* < 0.01) (Table 2). Therefore, all hypotheses (H1–H3) set to clarify construct validity have been proven.

**Fig. 1:**
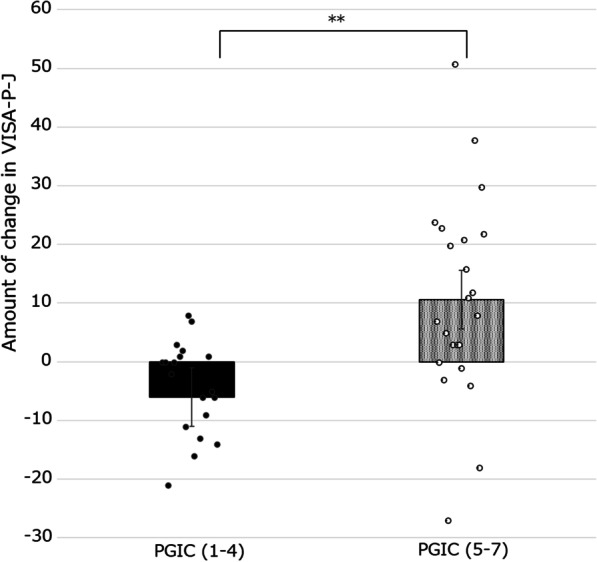
Change in VISA-P-J scores. The amount of change in the Victorian Institute of sports assessment for patellar tendinopathy (VISA-P-J) scores (12 weeks—baseline assessment) is the difference between the patient global impression of change (PGIC) 1–4 group and PGIC 5–7 group (*: *p* < 0.05;**p < 0.01)

Those who answered "5: minimally improved" to "7: very much improved" on the PGIC showed more improvement in the VISA-P-J (points) than those who answered "1: very much worse" to "4: no change" on the PGIC (*p* < 0.01) (Fig. 1). The amount of change in VISA-P-J scores showed a negative correlation between the amount of change in VAS-ADL and the amount of change in VAS-Sports (r = − 0.52, r = − 0.76, *p* < 0.01) (Table 2). Therefore, all the hypotheses (H4–H6) set to clarify responsiveness have been proven.

There was no floor or ceiling effect in the VISA-P-J, with < 15% of participants reporting minimum or maximum scores, respectively (Table 2). Eleven participants answered "5 = minimally improved" on the PGIC from the first measurement. The first measurement of the VISA-P-J that selected 11 participants was mean of 69.8 points, and the third measurement of the VISA-P-J was a mean of 76.8 points. Therefore, the MCID of the VISA-P-J for the 11 participants was seven (Table 2).

## Discussion

In the present study, we demonstrated that the VISA-P-J was valid, reliable, and responsive for individuals with pain in the inferior pole of the patella to the proximal patellar tendon for patellar tendinopathy.

0.87 for ICC of the VISA-P-J, which was higher than the minimum standard value [30] and, it is considered good reliability, which ranges between 0.75 and 0.9 range [32]. The ICC of the previous studies regarding the VISA-P cross-cultural adaptation ranged from 0.74 to 0.99 [8, 12]. The ICC for the test–retest reliability of the VISA-P-J was comparable to those of the previous studies [8, 9, 12, 13, 17]. The measurement error of the VISA-P-J was 0.89. A measurement error appears to be acceptable at < 10% and is considered appropriate for clinical and research purposes [33]. The Cronbach's alpha of the VISA-P-J is 0.81, within the 0.73–0.99 range, similar to that in the previous cross-cultural adaptation studies of the VISA-P [12, 18]. The methods of test–retest reliability according to the COSMIN guidelines are excellent and are considered higher than the standard value. The measurement error is < 10%. Cronbach’s alpha for internal consistency was near the maximum value of one [12]. Therefore, all the reliabilities showed high values, indicating excellent reliability.

The VISA-P-J assesses subjective symptoms in clinical and sports settings, such as patellar tendon pain, functions related to knee extension, and the ability to play sports [7]. To assess the validity of the VISA-P-J, a comparison with external standards that indicate symptoms in clinical and sports settings is required. Pain assessment using the VAS and symptom classification based on clinical symptoms were negatively correlated with the VISA-P-J scores. In addition, since more than 75% of the hypotheses (H1–H3) were substantiated [30], the VISA-P-J was considered valid in Japanese population.

A PGIC scale score of 5–7 is a positive change, while a PGIC scale score of 1–4 shows a negative change or no change. In this study, responsiveness was proven with a PGIC scale score of 5–7 as a more positive VISA-P-J score change than a PGIC scale score of 1–4. Additionally, it correlated with the amount of change in the VAS score. Only two studies have examined the responsiveness of the VISA-P in each language, the Spanish and Brazilian Portuguese versions [8, 9]. However, both studies did not use hypothesis-testing to demonstrate responsiveness. Since there is no gold standard for evaluating patellar tendinopathy, a hypothesis-testing method can be preferable [21]. Therefore, this study has an advantage and the responsiveness of VISA-P-J was considered valid.

The difference of seven points was calculated using the anchor method for the MCID of the VISA-P-J. The SEM was 0.89, and systematic and random errors in scores that occurred during measurement were below the MCID, suggesting that the current MCID is not within the error range and that the seven points represent a clinical change. The only previous study that examined the MCID of the VISA-P was the Spanish version, which was conducted on athletes ≥ 18 years old, and the results showed 13 points [23]. This study’s participants were athletes ≥ 18 years old, similar to the participants of the previous study. The baseline score in the previous study was 50.1, in which the symptoms were more severe than those in the present study. As the MCID has been reported to be dependent on the participant's initial score [34, 35], the difference in the initial score may affect the MCID score. Therefore, it is important to examine the MCID for the VISA-P-J in participants with different baselines.

In the present study, we should note some limitations. First, we only looked at PGIC changes of "5: minimally improved" and "4: no change" to calculate MCID. Also, we only calculated MCID by the anchor-based method based on an article by Katz NP et al. [31]. Therefore, the MCID may differ with the distribution-based method. Second, the number of participants was slightly small. In COSMIN, psychometric properties have their sampling evaluation criteria. Although the sample size of 43 is acceptable, it was not ideal. Lastly, the participants might receive some therapy during the study period. However, the details are unclear and are not available. Resolving this study’s limitation would allow the psychometric properties of the VISA-P-J to be evaluated as a higher standard.

## Conclusion

The VISA-P-J is a reliable, valid, and responsive assessment method for individuals with patellar tendinopathy in Japanese population based on the COSMIN.

## Supplementary Information

**Additional file 1.** The Japanese version of the Victorian Institute of sports assessment for patellar tendinopathy (VISA-P-J).

## Data Availability

The datasets generated and/or analysed during the current study are available from the corresponding author on reasonable request.

## Abbreviations

COSMIN: COnsensus-based Standards for the selection of health Measurement INstruments
VISA-P-J: Japanese version of the Victorian Institute of sports assessment scale for patellar tendinopathy
PROMs: Patient-reported outcome measures
ICC: Intraclass correlation coefficient
SEM: Standard error of measurement
VAS-ADL: Visual analog scale—activities of daily living
VAS-Sports: Visual analog scale—sports
PGIC: Patient global impression of change
MCID: Minimal clinically important difference
