# Supplementary material for: Cross-cultural adaptation, validity, reliability and responsiveness of the Japanese version of the Victorian Institute of sports assessment for patellar tendinopathy (VISA-P-J)
Source: BMC Sports Sci Med Rehabil. 2023 Jan 11;15:5. doi: 10.1186/s13102-023-00615-5 (PMC9832761; doi:10.1186/s13102-023-00615-5)
Supplement: Supplementary file 1 — Additional file 1. The Japanese version of the Victorian Institute of sports assessment for patellar tendinopathy (VISA-P-J). [file 13102_2023_615_MOESM1_ESM.pdf]

VICTORIAN INSTITUTE OF SPORT

日付：
名前：

1.痛みがない状態で、何分間座っていられますか？

□	□	□	□	□	□	□	□	□	□	□
0	10	20	30	40	50	60	70	80	90	100

単位=分

2.通常の歩行で階段を降りる時に、痛みはありますか？

□	□	□	□	□	□	□	□	□	□	□
0	1	2	3	4	5	6	7	8	9	10

非常に強い痛み=0、痛みなし=10

3.体重をかけていない状態で、自分の力で膝を真っ直ぐ伸ばした時に膝に痛みはありますか？

□	□	□	□	□	□	□	□	□	□	□
0	1	2	3	4	5	6	7	8	9	10

非常に強い痛み=0、痛みなし=10

4.完全に体重をかけたランジ動作で痛みはありますか？

□	□	□	□	□	□	□	□	□	□	□
0	1	2	3	4	5	6	7	8	9	10

非常に強い痛み=0、痛みなし=10

5.スクワット動作に問題はありますか？

☐	☐	☐	☐	☐	☐	☐	☐	☐	☐	☐
0	1	2	3	4	5	6	7	8	9	10

できない=0、問題なし=10

6.シングルレッグ・ホッピング（片脚ジャンプ）を 10 回行った場合、その最中や直後に痛みはありますか？

☐	☐	☐	☐	☐	☐	☐	☐	☐	☐	☐
0	1	2	3	4	5	6	7	8	9	10

非常に強い痛み／できない=0、痛みなし=10

7.現在、スポーツやその他の身体活動を行っていますか？

☐ 全くしていない（0）
☐ 負荷を制限したトレーニング± 患部への負荷を制限した競技に参加（4）
☐ すべてのトレーニング±競技に参加しているが、症状が出始めた時と同じレベルではない（7）
☐ 症状が出始めた時と同じか、それ以上のレベルで競技に参加（10）

8. この質問ではaからcのいずれかの質問に答えてください。

☐ スポーツをしている時に痛みがない場合→8aのみの質問に答えてください
☐ スポーツをしている時に痛みがあるが、活動を中止するほどでもない→8bのみの質問に答えてください
☐ スポーツ活動を完全に中止しなくてはならないほどの痛みがある→8c のみの質問に答えてください

8a.スポーツをしている間に痛みがない状態で、トレーニング／練習を何分間継続できますか？

☐	☐	☐	☐	☐
0分	1-5分	6-10分	7-15分	16分以上
0	7	14	21	30

8b.スポーツをしていると痛みが生じるが中止するほどでもない状態で、トレーニング／練習を何分間継続できますか？

☐	☐	☐	☐	☐
0分	1-5分	6-10分	7-15分	16分以上
0	4	10	14	20

8c.トレーニング／練習を中止しなくてはならないほどの痛みが生じる場合、トレーニング／練習を何分間継続できますか？

☐	☐	☐	☐	☐
0分	1-5分	6-10分	7-15分	16分以上
0	2	5	7	10

合計スコア__________
